# Regional-scale monitoring of underwater and dry ground subsidence in high phreatic areas of North China Plain

**DOI:** 10.1371/journal.pone.0237878

**Published:** 2020-08-24

**Authors:** Jingjing Zhou, Young Gu Her, Beibei Niu, Maosen Zhao, Xinju Li, Xinyang Yu

**Affiliations:** 1 College of Resources and Environment, Shandong Agricultural University, Tai’an, China; 2 UF/IFAS Tropical Research and Education Center, University of Florida, Homestead, Florida, United States of America; 3 Information Center, Taishan Sanatorium of Shandong Province, Tai’an, China; Shenzhen University, CHINA

## Abstract

Land subsidence monitoring provides information required when developing land use plans and allows for proactive management of subsidence issues. However, it has been challenging to accurately detect land subsidence areas, especially those under waterbodies. This study evaluated the applicability of integrated use of the optical Landsat-8 OLI and microwave Sentinel-1A TOPSAR imagery to delineate subsidence areas and quantify subsidence rates in a typical coal mining area of North China Plain. An Enhanced Modified Normalized Difference Water Index (E-MNDWI) was combined with Short BAseline Subset-Interferometric Synthetic Aperture Radar (SBAS-InSAR) image to monitor underwater and dry ground subsidence. The results demonstrated that the method could delineate underwater and dry ground subsidence and quantify its rates accurately. The proposed method estimated subsidence area corresponded to 34.8% (16.7 km^2^) of the study area. The size of underwater subsidence areas was substantial and accounted for 43.7% of the subsidence areas. Seasonal underwater subsidence areas were generally distributed in the vicinity of perennial ones. Dry ground subsidence covered 9.4 km^2^ of the study area and generally occurred in urban and rural residential areas with the maximum subsidence of up to 80.1 mm/year. This study demonstrates the efficiency and capacity of integrating optical and microwave images to monitor the subsidence progresses, which thus can help develop effective rehabilitation policy and strategy to mitigate the impacts of land subsidence.

## Introduction

Land subsidence can occur on underwater and dry ground surfaces [1]. Coal mining areas in high phreatic region are prone to subsidence due to intensive mining activities [2,3]. However, the lack of tools and methods for monitoring and predicting subsidence has prevented the proactive planning and management for areas with high subsidence risk. Accurate detection and delineation of land subsidence are of prime importance to maintain the local sustainability and economic development.

Studies have used geodetic leveling and Global Positioning System (GPS) to detect land subsidence [4,5]. However, the field survey-based methods were labor-intensive and time-consuming, and thus they would be inefficient for monitoring land subsidence and its spatial distributions in large areas [6,7]. Remotely sensed imagery and associated analysis techniques have attracted attention as an efficient way to monitor environmental issues including subsidence as well as land use changes [8–10]. The Spaceborne Synthetic Aperture Radar (SAR) systems and datasets have provided information useful to environmental planning and management since the 1980s [11]. The Sentinel-1A satellite provides continuous terrain observations with progressive scans SAR (TOPSAR) images at a high spatial resolution regardless of weather conditions [12,13]. TOPSAR enables the Extra Wide (EW) and Interferometric Wide Swath (IW) modes and facilitates interferometric SAR [14,15]. In these modes, bursts are synchronized from pass to pass to ensure the alignment of interferometric pairs, which shows advantages in-ground motion monitoring, cryosphere dynamics, forest, and soil mapping and management [16].

The Interferometric Synthetic Aperture Radar (InSAR) technique has improved rapidly since it was proposed, and it is now one of the most efficient and accurate millimeter-scale remote sensing technologies, especially for land subsidence monitoring [17]. There are several InSAR techniques for the analysis of SAR imagery, and SBAS-InSAR technique is one of the most widely used methods. The SBAS-InSAR technique can effectively adjust space-time disassociation and atmospheric delays [18], and it showed great potential in detecting long-term cumulative and spatially continuous subsidence [19]. The SBAS-InSAR and Sentinel-1A TOPSAR images have been successfully used in monitoring ground deformation in urban area subsidence [20,21], landslide detecting in Loess Plateau [22] and permafrost freeze-thaw seasonal displacement over the Qinghai-Tibetan Plateau [23]. Due to the ever-changing hydro-meteo-geological characteristics and the shortage of supporting data, few studies investigated the land subsidence in high phreatic region. The potential of the combined use of SBAS-InSAR and Sentinel-1A TOPSAR images has not been evaluated to detect and monitor on-going land subsidence. The information of land subsidence is necessary for developing sustainable landscape management and restoration plans.

Although SAR imagery has many advantages, it was known that the imagery does not accurately distinguish the boundaries between waterbodies and dry grounds due to Doppler shift effects associated with the gravity wave orbital motions and low coherence associated with water [24,25]. SAR imagery alone is not qualified to monitor the land subsidence situation in watery areas. Studies used Landsat-8 OLI images and water indices to improve the accuracy of delineating the boundaries of water areas [26,27]. Although Landsat-8 OLI images have not been used to detect underwater subsidence, the combination of Landsat-8 OLI images and high-efficiency water index has the potential to accurately identify underwater ground areas where subsidence is progressing. It is essential to investigate how the integrated use of the different types of images can help quantify subsidence in an area with complicated landscape.

This study explored and proposed ways to delineate the areas of subsidence on underwater and dry ground surfaces and estimate its scope and rate using optical Landsat-8 and microwave Sentinel-1A TOPSAR images. The proposed subsidence detection method was applied to the Xinglongzhuang coal mining area of North China Plain to demonstrate its efficacy. From this study, we investigated the accuracy of Landsat-8 OLI and Sentinel-1A TOPSAR images in detecting subsided areas on underwater and dry ground surfaces and the spatiotemporal variation of areas where subsidence is progressing in the study region.

## Materials and methods

### Study region

**Fig 1:**
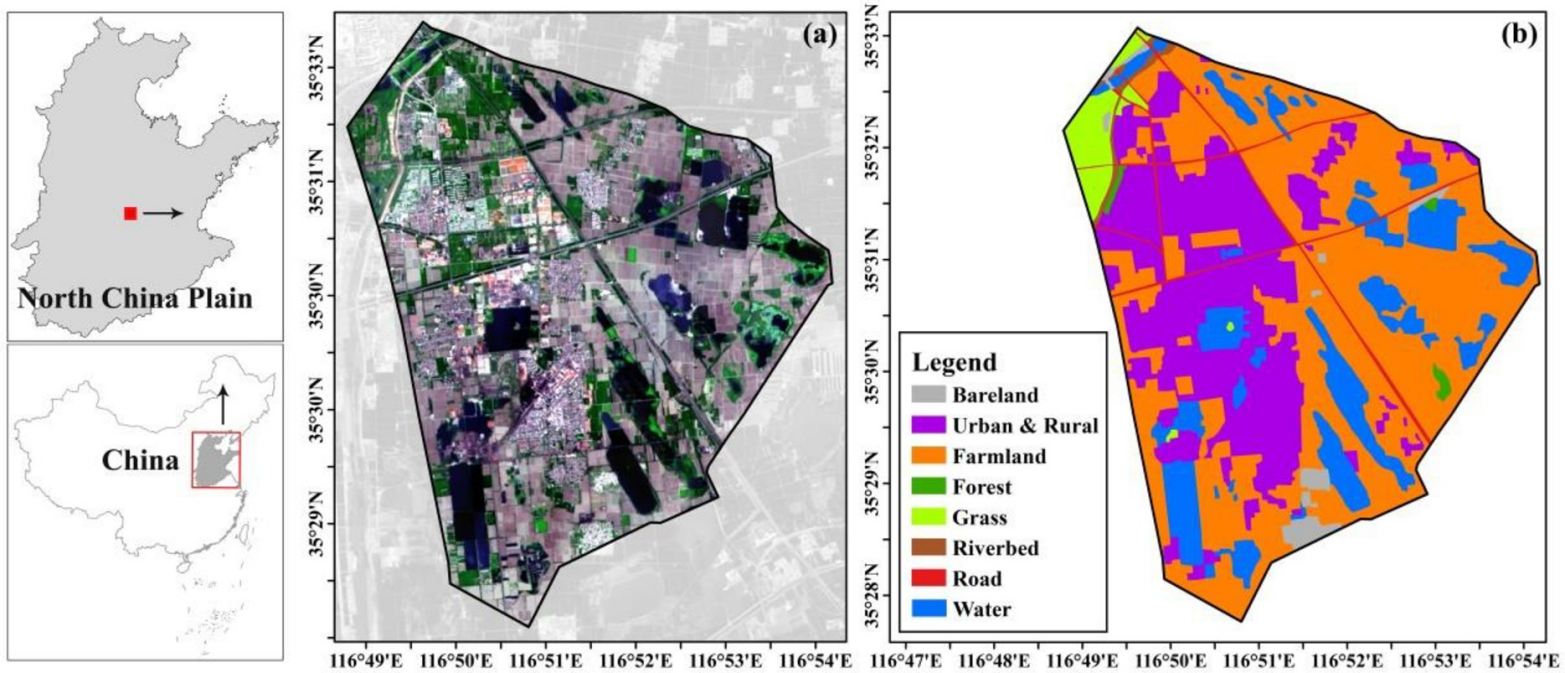
Location and land use map of the study area. (a): Location of the study area, the black line represents the boundary of the coal mining area on a Landsat-8 OLI image (the false color composition) obtained from the Earth Resources Observatory and Science (EROS) Center (http://eros.usgs.gov) in June 2017. (b): Land use map of the study area. The land uses were interpreted visually based on the Landsat-8 OLI image of June 2017and Google Earth^®^ high-resolution image.

The Xinglongzhuang region (116°49′E to 116°54′E, 35°26′N to 35°33′N, 48 km^2^) is located in the high phreatic region of North China Plain (Fig 1A). The region mainly consists of farmland (48.5%), urban and rural residence areas (28.3%), and waterbody (17.1%) (Fig 1B). The large seasonal variations of precipitation and temperature lead to apparent temporal changes in the soil water and groundwater dynamics, which make the boundary of subsidence substantially expand or shrink over seasons in the study area. The depth of groundwater in the study area ranges from 2.0 to 2.5 m, and land subsidence is the main cause of creating ponded water areas [28–30]. The coal mining facilities that were put into operation in 1981 have been exacerbating the subsidence issue [31]. The hydro-meteo-geological characteristics and mining activities form subsidence zones that are covered by water perennially or seasonally, which made the study area ideal to evaluate the efficacy of land subsidence monitoring methods.

### Remotely sensed imagery and auxiliary information

The multi-temporal Landsat-8 OLI image was employed to extract and monitor the perennial/seasonal underwater ground subsidence areas. Dry ground subsidence areas were separated from underwater ones using Sentinel-1A TOPSAR data. Specifically, Landsat-8 OLI images acquired in summer and winter seasons including July and December of 2015, July and December of 2016, and June of 2017, which have cloud-free fine image quality, were selected to extract the perennial/seasonal underwater ground subsidence areas (no data available in July 2017) (Table 1). Landsat-8 OLI Images obtained for individual months from the Earth Resources Observation and Science Center (EROS) showed that the extents of waterbodies usually reach their maximum and minimum levels in summer and winter, respectively.

**Table 1:** Metadata of Landsat-8 OLI images covering the study region. The spatial resolutions of images were 30 m for multi-spectral bands and 15 m for the panorama band.

Year	Path/Row	Landsat Scene ID	Acquisition Date (mm-dd-yyyy)	Spatial Resolution (m)
2015	122/35	LC81220352015195LGN00	07-14-2015	30/15
2015	122/35	LC81220352015355LGN01	12-21-2015	30/15
2016	122/35	LC81220352016198LGN00	07-16-2016	30/15
2016	122/35	LC81220352016342LGN01	12-07-2016	30/15
2017	122/35	LC81220352017168LGN00	06-17-2017	30/15

A total of 23 Sentinel-1A TOPSAR data (C-band) covering the study area, which were captured between 20 July 2015 and 16 June 2017 and obtained from Copernicus Open Access Hub of European Space Agency (https://scihub.copernicus.eu/dhus/#/home), were selected to estimate vertical average subsidence rate and track its temporal variations on dry grounds (Table 2). The specification of the SAR images were as follows: track number 142, central incidence angle on the test site 41.9°, Slant range/Azimuth resolution 2.3/13.9 m, and acquired in the ascending orbit with a VV polarization. The 3 arc-second Shuttle Radar Topography Mission (SRTM) digital elevation model (DEM) provided by the National Aeronautics and Space Administration (NASA) was adopted to remove topographic phases. Precise Orbit Determination (POD) data released by ESA were used for the orbital refinement and phase re-flattening. Multiple reference data and ground truths, including the land use maps of 1980 and 1985, geodetic leveling data (May 2016 to March 2017), and data from field survey implemented in July 2017 were employed to delineate underwater ground subsidence areas and verify the estimates.

**Table 2:** Statistics of underwater ground subsidence areas.

Year	Underwater ground subsidence area (km^2^)	
	Wet season	Dry season
2015	6.6	6.0
2016	6.8	6.7
2017	7.3	-

### Data processing

#### Landsat-8 OLI images

Radiometric calibrations and atmospheric correction using Fast Line-of-sight Atmospheric Analysis of Hypercubes [32] were conducted to the selected Landsat-8 OLI images. The Nearest Neighbor Diffusion Based Pan Sharpening Algorithm was performed to the images to improve the spatial resolution of multispectral bands to 15 m [33]. Field survey found that the coal mining was generally piled up on the storage yard in the open air, the storage place would have the same index value range with waterbody when using other water indices such as MNDWI, which may lead to overestimation results. To better identify the boundary of waterbodies, an enhanced modified normalized difference water index (E-MNDWI, Eq (1)) was implemented to the five Landsat-8 images. The second shortwave band was employed in the equation because from the insitu field study we found that the second shortwave is sensitive to coal and mining waste, which can be used to identify waterbody from coal storage place [34]. The Iterative Self-Organizing Data Analysis Technique (ISODATA) method was then applied to the five E-MNDWI images so that water areas can be delineated in a more objective manner solely based on the images and their spectral characteristics [35]. The results of ISODATA were then reclassified into two classes: underwater ground and dry ground areas. The delineated water-covered areas included natural waterbodies (e.g., rivers, natural lakes) and underwater ground subsidence areas that have been expanded by coal mining activities since 1981.

$$E-MNDWI=(\rho_{Green}-\rho_{\sum SWIR})/(\rho_{Green}+\rho_{\sum SWIR})$$ (1)

where *ρ_Green_* is the reflectance of green band (band 3) of Landsat-8 OLI image, *ρ*_∑*SWIR*_ represents reflectance summation of the two shortwave infrared bands (band 6 and band 7 of Landsat-8 OLI image).

To separate underwater subsidence areas from natural waterbodies, historical land use maps of 1980 and 1985, which were surveyed and mapped from field investigation before and after the coal mining activity, were employed to identify natural waterbodies that existed before the coal mine. After removing the natural waterbodies from the E-MNDWI analysis results, waterbodies that were created due to subsidence were further analyzed using a dynamic change analysis method. Underwater areas identified in the dry seasons were regarded as perennial underwater subsidence areas, and the other underwater areas in the wet season were classified as seasonal underwater subsidence areas.

#### Sentinel-1A TOPSAR imagery

**Fig 2:**
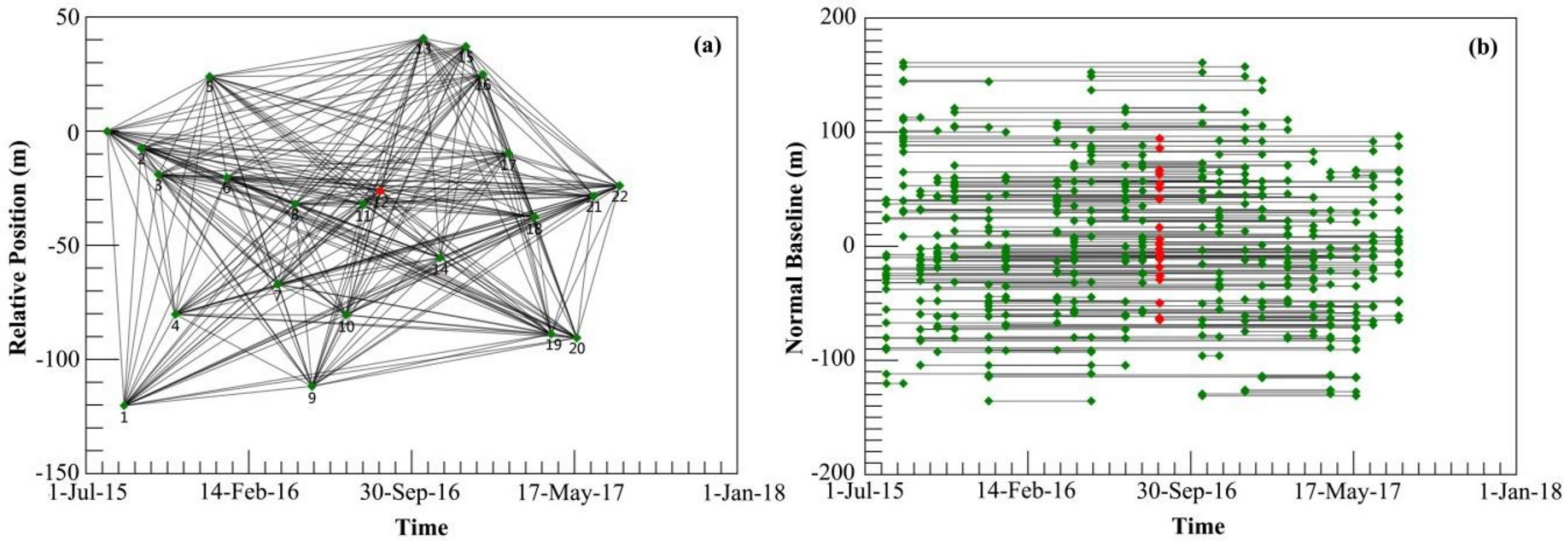
Relative position and time baseline of the imagery. (a) Relative position of interferometric pairs; (b) time-baseline interferometric pairs. The red diamond is the super master image. Black lines represent interferometric pairs. Green dots denote slave images.

The SARscape^®^ Modules (Version 5.1) for ENVI 5.3 software suit was employed to apply SBAS-InSAR and perform interferometric analyses. The Sentinel-1A TOPSAR image acquired on 17 August 2016, was selected as the master image for the interferometric combinations, and all slave images were co-registered and resampled to the super master image (Fig 2). The Delauney 3D method was employed for phase unwrapping with an unwrapping coherence threshold of 0.35 [36]. Then, we processed the original Sentinel-1A TOPSAR images such as flattening, filtering interferograms and unwrapping phases to remove potential errors that might be caused by orbit inaccuracy, non-coherent pairs, atmospheric artifacts, and residual topography. In the reflattening process, this study selected 24 reference points whose unwrapped phase values close to zero on flat areas identified from a reference topographic map [37]. Finally, geocoding was performed in the original satellite line of sight direction with a pixel resolution of 20 m.

### Verification

The agreement between land subsidence rates observed from the July 2017 field survey and predicted using the remotely sensed datasets was quantified using the coefficient of determination (*R*^2^) and *RMSE*.

$$RMSE=\sqrt{(\sum_{i=1}^{n}{\left(\Delta H_{i}-\Delta h_{i}\right)}^{2})/n}$$ (2)

$$R^{2}=1-(\sum_{i=1}^{n}{\left(H_{i}-h_{i}\right)}^{2})/(\sum_{i=1}^{n}{\left(H_{i}-\bar{h_{i}}\right)}^{2})$$ (3)

where *RMSE* is the root mean square error, and *R*^2^ represents the coefficient of determination. *H_i_* represents the insitu reference data, *h_i_* is the estimated value, $\bar{h_{i}}$ is the average estimated value, and n represents the sum number of reference data. In the field survey, 15 underwater grounds were randomly selected in the study region (Fig 4A), and their areas were measured by walking along their boundaries with a hand-held GPS. The average subsidence velocity data from 33 leveling points distributed in the study area were obtained from the Geological Engineering Investigation Institute of South Shandong Province. The data were acquired from May 2016 to March 2017, and they were used for the verification of dry ground subsidence estimates in this study. The subsidence velocity based on the SBAS-InSAR method in the same period was extracted and correlated with the leveling data using the leave-one-out cross validation [38].

## Results

### Verification of subsidence detection

**Fig 3:**
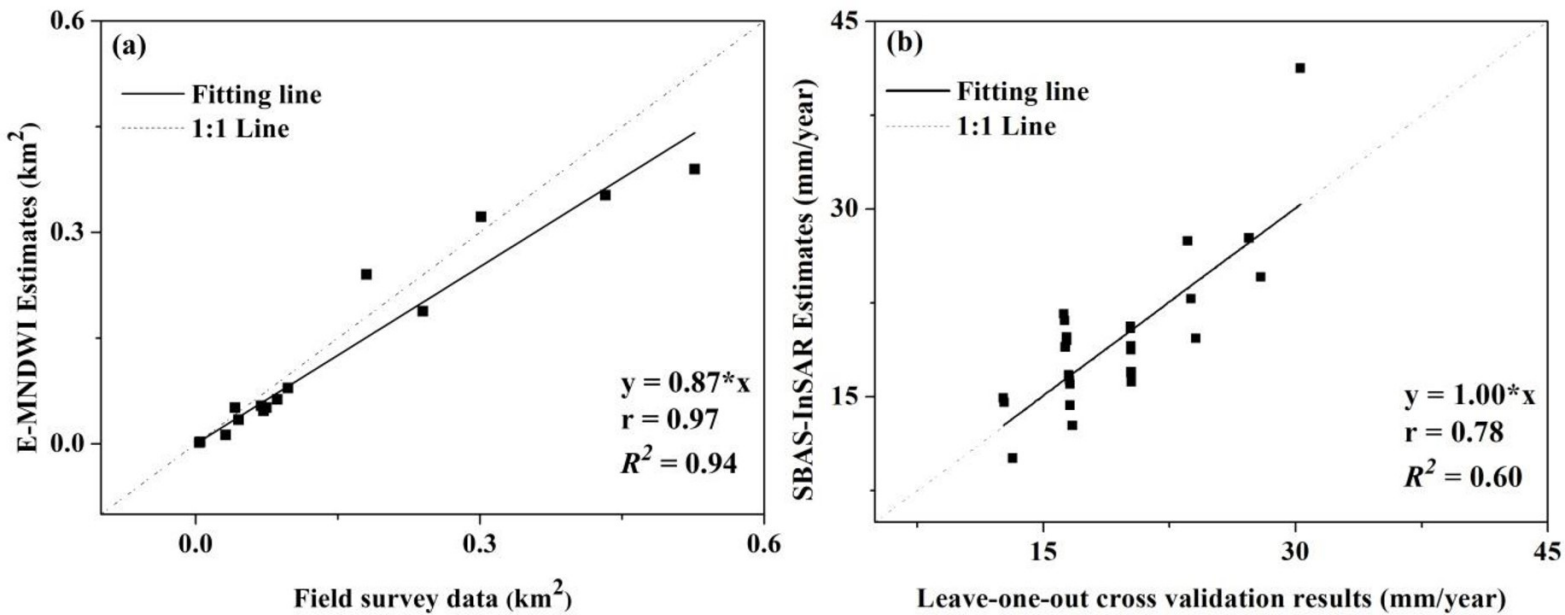
Agreement between observed and estimated underwater subsidence areas (a) and dry ground subsidence rates (b). Black solid lines are the fitting results, and grey dash ones are the 1:1 lines.

**Fig 4:**
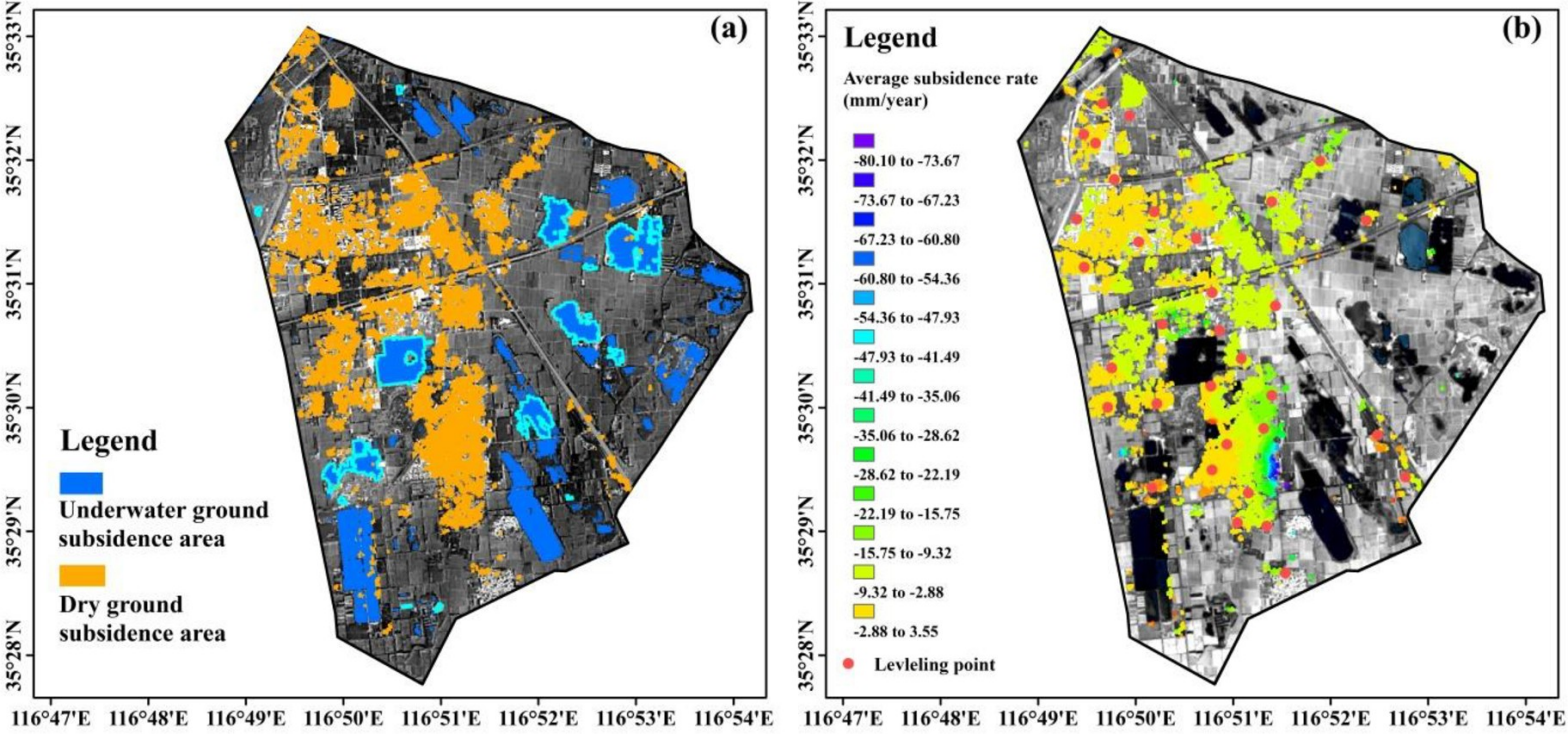
Subsidence area identification using the optical and microwave remote sensing images. (a): Underwater and dry ground subsidence areas, the 15 highlighted waterbodies were used for verifying the subsidence rate estimates. (b): Estimated average subsidence rates in the study area; red dots denote the locations of leveling points. The analysis results were overlaid on the Landsat-8 OLI image of June 2017, which was obtained from the public domain: http://eros.usgs.gov of the Earth Resources Observatory and Science (EROS) Center.

The areas of underwater ground subsidence identified from the E-MNDWI images were compared with the field survey data. E-MNDWI estimates were highly correlated to the field measurements, while it slightly underestimated underwater ground subsidence areas consistently (Fig 3A). The underestimation might be attributed to the existence of mixed pixels in the Landsat-8 OLI images. The accuracy of the dry ground subsidence monitoring was verified by comparing the estimates made using the Sentinel-1A TOPSAR data and SBAS-InSAR method with the results of the ground surface level survey performed at the 33 leveling points (Figs 3B and 4B). The correlation structure between the SBAS-InSAR extracted subsidence velocity and the leave-one-out cross validation results was relatively strong with the correlation coefficient value of 0.78 and the average *RMSE* of 3.38 mm/year. Such comparison and cross validation results demonstrated the potential of E-MNDWI and the SBAS-InSAR method as an affordable but precise tool to monitor underwater subsidence areas and dry ground subsidence rates over time.

### Subsidence delineation

The spatial extents of the underwater ground and dry ground subsidence identified using the remotely sensed data and analysis techniques were depicted in Fig 4. It could be found that the underwater ground subsidence distributed relatively evenly in the study region, while the dry ground subsidence mainly occurred in the west (Fig 4A). The total area of subsidence occurred during the study period was 16.7 km^2^, which corresponded to 34.8% of the study area, indicating the severity of the subsidence issue in the study region.

**Fig 5:**
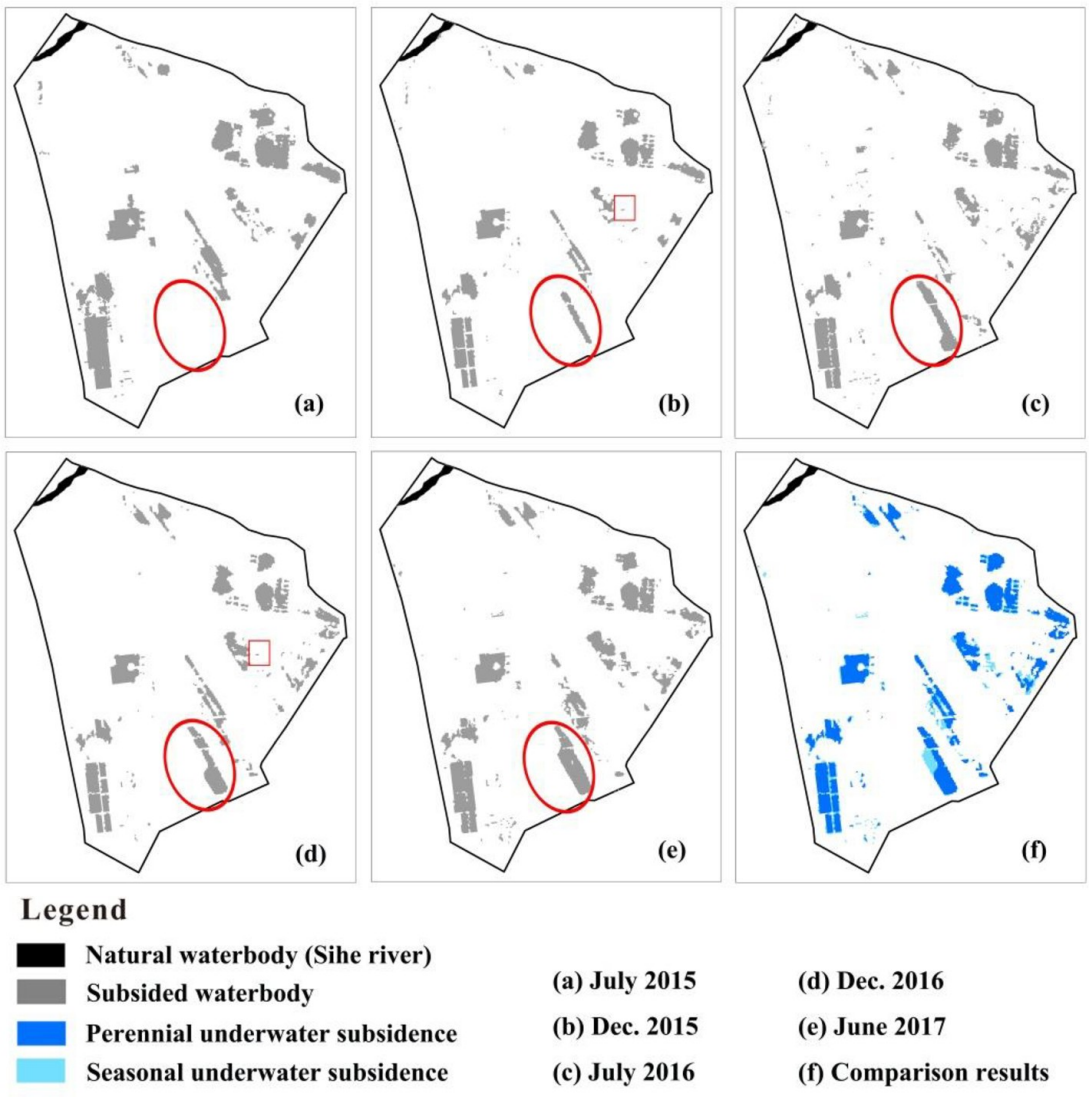
Underwater ground subsidence areas estimated from Landsat-8 OLI images. The image was obtained from the public domain, http://eros.usgs.gov, of the EROS Center. E-MNDWI values on (a) December July 2015, (b) December 2015, (c) July 2016, (d) December 2016, (e) June 2017, and (f) the comparison between perennial and seasonal underwater ground subsidence areas. The red eclipse and rectangle indicated the location of WP-A and WP-B, respectively.

The Landsat-8 OLI image analysis conducted with the E-MNDWI showed that a large part (43.7%, 7.3 km^2^) of the subsidence areas was occurred under water, indicating the significance of underwater ground subsidence (Table 2). The image analysis found that the spatial extents of underwater ground subsidence were expanded in wet (or summer) seasons and shrunk in dry (or winter) seasons (Fig 5A–5E and Table 2). The seasonal underwater ground subsidence areas were found to be generally distributed in the vicinity of perennial ones (Fig 5F). During the wet seasons, subsidence areas under ponded water increased by 2.9% from 2015 to 2016 and 7.4% from 2016 to 2017. When focusing on a water pit (WP-A hereafter) formed in December 2015 (red ellipse area in Fig 5A–5E) as an example, the sizes of subsided underwater areas grew from zero in July 2015 to 0.4 km^2^ in July 2016 (Fig 5C) and then further extended to 0.6 km^2^ in June 2017 (Fig 5E). The newly increased area was mainly located in the south part of WP-A. In the dry seasons, the underwater subsidence area increased by 11.7% (Table 2). WP-A covered an area of 0.2 km^2^ in December 2015 and 0.5 km^2^ in December 2016, and most of the increases in the underwater subsidence areas happened in WP-A during the study period. The annual variation of the other perennial water areas was not substantial. For instance, a field-surveyed waterbody (denoted as WP-B, a red rectangle in Fig 5B–5D) covered the same area of 0.003 km^2^ in December 2015 and December 2016.

The dry ground subsidence areas were delineated from the Sentinel-1A TOPSAR images using the SBAS-InSAR method. Based on leveling data, the location point of 30.506° N and 116.865° E was selected as a reference point on which the subsidence rates were calculated [39,40]. Negative and positive signs of the subsidence rates represent the depression and uplift of the ground surface in the vertical direction, respectively. Unlike the case of underwater ground subsidence areas, dry ground subsidence generally occurred in urban and rural residential areas, and most of the subsided dry ground surfaces were located in the west of the study region (Fig 4B). During the monitoring period, subsidence occurred in the dry areas of 9.4 km^2^, which accounted for 69% of the total urban and rural residence areas. The land subsidence partially covered each of the administrative units in the study area. The dry area subsidence rates reached 80.1 mm/year in the northwest of WP-A during the study period. Such finding was consistent with the estimates made using the multi-temporal E-MNDWIs. For instance, the subsidence on the dry ground surface was substantially expanded in WP-A.

## Discussion

After more than thirty years’ development, land subsidence has become one of the most severe socio-economic and environmental issues of North China Plain [41,42]. This study first separately investigated and quantified underwater and dry ground subsidence and demonstrated the significance of underwater subsidence using optical and microwave remotely sensed images. The results demonstrated that the use of Landsat-8 OLI and Sentinel-1 TOPSAR combined with the E-MNDWI and SBAS-InSAR methods could accurately identify subsidence areas and estimate the subsidence rates, which provide a scientific basis for developing subsidence management plans and practices. The results also showed that substantial land subsidence is still going on, and it could be associated with the mining activities in the study area. The results suggest closely monitoring subsidence and developing efficient policies to mitigate subsidence and its impacts.

The Landsat-8 OLI multispectral images with historical land use maps provided essential spectral information of the study area that can help distinguish underwater subsidence areas. WP-A occupied most of the expanded underwater subsidence area. The analysis of Sentinel-1A TOPSAR images also found that the dry ground area in the adjacent areas of WP-A suffered apparent land subsidence (Fig 4B). The combination of Landsat-8 OLI and Sentinel-1A TOPSAR images could precisely detect and monitor land subsidence in high phreatic region. Due to the limitation of optical sensing techniques and mechanisms (limited ability of penetrating water), the Landsat-8 images (unlike the SAR images) did not provide information that we could use to estimate the subsidence rates, which is a disadvantage of using such types of images. In addition, the identification of underwater subsidence areas was performed at the resolution of 15 m, which is equivalent to the resolution of the Landsat layers. The application of images that have higher spatial resolutions is expected to be able to provide more precise subsidence areas delineation, which however was left for the following study.

## Conclusion

This study combined optical and SAR images to delineate underwater and dry ground subsidence areas in a typical high phreatic region of North China Plain. The results demonstrated that the use of Landsat-8 OLI and Sentinel-1A TOPSAR combined with the E-MNDWI and SBAS-InSAR methods could identify subsidence areas and estimate the subsidence rates accurately. The Landsat-8 OLI multispectral images with historical land use maps provided essential spectral information of the study area that can help identify underwater subsidence areas. Dry ground subsidence occurred in both urban and rural residential areas in the case study. This study also showed that substantial land subsidence is still going on, especially in areas close to coal mines in the study area, and further studies focusing on subsidence monitoring using remotely sensed images with higher spatial resolutions is recommended.

## Supporting information

S1 File Shapefiles of the study area and land use classification. (ZIP)

S1 Dataset Underwater subsidence data. (ZIP)

S2 Dataset Dry ground subsidence data. (ZIP)
